# Dietary and Physical Activity Habits of Children and Adolescents before and after the Implementation of a Personalized, Intervention Program for the Management of Obesity

**DOI:** 10.3390/nu16203477

**Published:** 2024-10-14

**Authors:** Georgia Ioannou, Ioulia Petrou, Maria Manou, Athanasia Tragomalou, Eleni Ramouzi, Aikaterini Vourdoumpa, Sofia-Maria Genitsaridi, Athanasia Kyrkili, Christos Diou, Marina Papadopoulou, Penio Kassari, Evangelia Charmandari

**Affiliations:** 1 Division of Endocrinology, Metabolism and Diabetes, First Department of Pediatrics, National and Kapodistrian University of Athens Medical School, ‘Aghia Sophia’ Children’s Hospital, 11527 Athens, Greece; geoioan95@gmail.com (G.I.); tzoulia.petrou@gmail.com (I.P.); mariamanou93@hotmail.com (M.M.); nansymou@hotmail.com (A.T.); eleni_ramouzi@hotmail.gr (E.R.); katvourdouba@gmail.com (A.V.); sgenitsaridi@gmail.com (S.-M.G.); athanasiakirkili@gmail.com (A.K.); marinageorpap@gmail.com (M.P.); peniokassari@gmail.com (P.K.); 2 Department of Informatics and Telematics, Harokopio University of Athens, 17671 Athens, Greece; cdiou@hua.gr; 3 Center of Clinical, Experimental Surgery and Translational Research, Biomedical Research Foundation of the Academy of Athens, 11527 Athens, Greece

**Keywords:** childhood obesity, diet, physical activity, big data, smartphone, smartwatch, lifestyle intervention

## Abstract

Background: Obesity in childhood and adolescence represents a major public health problem, mostly attributed to dietary and physical activity factors. We aimed to determine the dietary and physical activity habits of participants before and after the implementation of a personalized, multidisciplinary, lifestyle intervention program for the management of obesity in the context of the Horizon Research Project ‘BigO: Big Data against Childhood Obesity’. Methods: Three hundred and eighty-six (n = 386) children and adolescents (mean age ± SD: 12.495 ± 1.988 years, 199 males and 187 females) participated in the study prospectively. Based on body mass index (BMI), subjects were classified as having obesity (n = 293, 75.9%) and overweight (n = 93, 24.1%) according to the International Obesity Task Force (IOTF) cut-off points. We implemented a personalized, multidisciplinary, lifestyle intervention program providing guidance on diet, sleep, and exercise, and utilized the BigO technology platform to objectively record data collected via a Smartphone and Smartwatch for each patient. Results: Following the intervention, a statistically significant decrease was noted in the consumption of cheese, cereal with added sugar, savory snacks, pasta, and fried potatoes across both BMI categories. Also, there was an increase in daily water intake between meals among all participants (*p* = 0.001) and a reduction in the consumption of evening snack or dinner while watching television (*p* < 0.05). Boys showed a decrease in the consumption of savory snacks, fried potato products, and pasta (*p* < 0.05), an increase in the consumption of sugar-free breakfast cereal (*p* < 0.05), and drank more water between meals daily (*p* < 0.001). Conclusions: Our findings suggest that a personalized, multidisciplinary, lifestyle intervention improves the dietary habits of children and adolescents.

## 1. Introduction

Since 1997, the World Health Organization (WHO) has officially recognized obesity as a global epidemic and a significant public health concern [1]. According to the WHO, in 2016, more than 1.9 billion adults were overweight, with over 650 million classified as having obesity. Similarly, childhood and adolescent obesity rates have seen a notable increase in recent decades. The WHO reports that, in 2020, 39 million children under the age of five years had overweight or obesity, while in 2016, over 340 million children and adolescents aged 5–19 years were classified as having overweight or obesity [2]. Greece is one of the main countries in Europe where the prevalence of childhood obesity has risen rapidly during the last four decades [3].

Overweight and obesity in childhood and adolescence is associated with increased morbidity and mortality, and poses a significant economic burden on society and the healthcare system, with both direct and indirect impacts [1,4,5,6,7,8]. According to the WHO, at least 2.6 million deaths annually are due to overweight or obesity. In addition, children and adolescents with overweight and obesity are likely to have overweight and obesity in adulthood and to develop non-communicable diseases, such as diabetes mellitus type 2 (DM2) and cardiovascular disease at an early age [9].

The primary cause of overweight and obesity in childhood and adolescence is an energy imbalance, characterized by an excess of calorie intake over expenditure, and can be attributed to specific factors. In terms of dietary habits, there has been a global increase in the intake of energy-dense foods that are high in fat and sugars and low in vitamins, minerals and other micronutrients. In addition to dietary habits, the rise in childhood and adolescent obesity is also attributed to the increasing prevalence of sedentary behavior, leading to reduced physical activity [2]. There is growing evidence indicating that children and adolescents are not meeting the recommended levels of daily physical activity [10,11].

Numerous interventions have been implemented over the years to address the epidemic of childhood and adolescent obesity, ranging from school-based initiatives to behavioral programs administered by multidisciplinary teams [12,13,14]. However, while some of these interventions have demonstrated effectiveness, they are often “inconvenient”, expensive, or not accessible to patients. Consequently, there is a growing demand for innovative and creative interventions [14,15].

Technological advances have facilitated the development of e-Health interventions aimed at preventing and treating childhood obesity. These interventions include various interactive platforms, real-time feedback, smartphone applications, interactive video games, mobile phone messaging services, and smartwatches with or without sensors. They can exist independently or as part of a comprehensive treatment. These technological tools primarily focus on improving dietary habits, enhancing physical activity levels, and increasing the user’s self-control and self-monitoring capacity [14,16].

An example of an innovative e-Health application is the HORIZON program ‘BigO: Big Data against Childhood Obesity’, which collects big data and allows conclusions to be drawn about the behavioral causes of childhood obesity. Specifically, the project aims to collect and analyze comprehensive data on the behavior and environment of children and adolescents with overweight and obesity, in order to enable public health decision-makers to design effective interventions to manage childhood obesity. The system includes the BigO technology platform which interfaces with smartphones and smartwatches (optionally), capturing objective data (using inertial sensors and GPS) for each participant, such as physical activity levels, sleep patterns, and user-reported metrics (e.g., pictures of meals, mood evaluation, etc.). These data are then transmitted to servers for analysis, extracting information related to dietary habits, physical activity and the environment of the children and adolescents [17]. The data are processed using Apache Spark to extract behavioral and environmental indicators and perform statistical analysis and machine learning tasks. The specific hypothesis underlying this program is that there are identifiable associations between lifestyle and environmental conditions and obesogenic behaviors, which can be quantified and modeled. This hypothesis guides the data collection and processing strategy, as well as the choice of statistical and machine learning models used for analysis [18].

In our study, we aimed to fill a gap in the scientific literature by broadly, thoroughly, and objectively recording and evaluating the lifestyle habits of children and adolescents receiving personalized, multidisciplinary, lifestyle intervention for the management of overweight and obesity. Consequently, the objective of this study was to record and assess the dietary and exercise habits before and after the implementation of the lifestyle intervention program in children and adolescents aged 8-18 years, who participated in the BigO program. We demonstrated that the implementation of our multidisciplinary, lifestyle intervention program resulted in significant improvement in the participants’ dietary and physical activity habits. We expect that these positive outcomes will contribute to the formulation of health policies targeting the management of childhood and adolescent obesity, thus enhancing initiatives aimed at preventing and treating this epidemic.

## 2. Materials and Methods

### 2.1. Study Design and Study Sample

**Figure 1:**
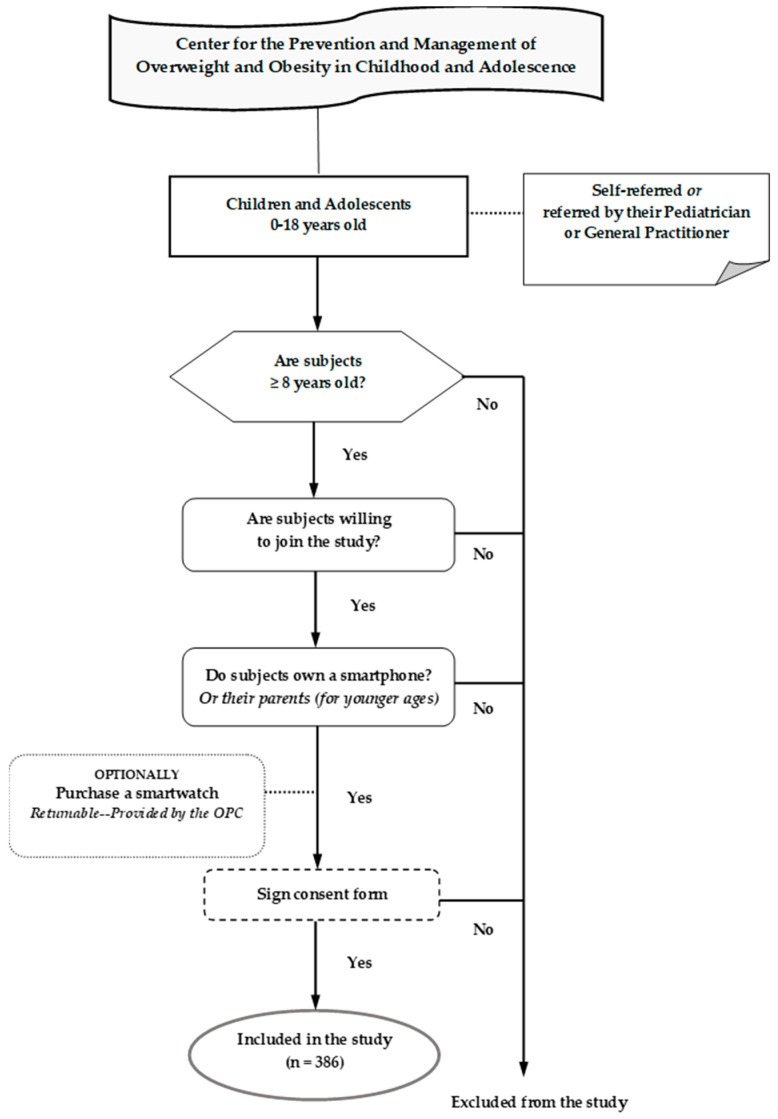
Flow chart of the participant selection process.

Our research study was a prospective cohort study, focusing on the dietary and exercise habits of children and adolescents. These habits were evaluated and analyzed upon entry into the study and again 12 months after the lifestyle intervention. The study involved children and adolescents aged 8–18 years, who were consecutive attendees at the Center for the Prevention and Management of Overweight and Obesity in Childhood and Adolescence, Division of Endocrinology, Metabolism and Diabetes, First Department of Pediatrics, National and Kapodistrian University of Athens Medical School, “Aghia Sophia” Children’s Hospital. All subjects were self-referred or referred by their pediatricians or general practitioners, they were motivated to reduce their BMI, and were interested in attending our Outpatient Clinic. The conditions for participation were their willingness to join the study, owning a smartphone (or their parents for younger ages), optionally purchasing a smartwatch if desired (returnable—provided by the clinic), and signing the consent form. Figure 1 presents the flow chart outlining the participants selection process. In total, 386 children and adolescents participated in the study, completing questionnaires both at the beginning of the study (initial assessment) and after one year of the implementation of the lifestyle intervention program (annual assessment).

### 2.2. Assessment and Lifestyle Intervention

All participants were evaluated by a pediatrician, pediatric endocrinologist, pediatric dietician, professional fitness personal trainer, and, when necessary, a pediatric clinical psychologist. At initial assessment, subjects were admitted to the Division of Endocrinology, Metabolism and Diabetes on the morning of the day of their initial visit, where they underwent a medical assessment by a pediatrician and a pediatric endocrinologist. This assessment included a detailed medical history and physical examination. Vital signs, such as blood pressure and heart rate, were recorded, along with anthropometric measurements, including weight, height, waist circumference, and hip circumference.

Weight was measured for each child in light clothing and without shoes, using a standardized scale (Seca, Hamburg, Germany). Measurements were recorded in kilograms to the nearest decimal place (0.1 kg). Height was determined without footwear, using the same calibrated stadiometer (Harpender/Holtain Limited, Crymych-Dyfed, UK), with height recorded in centimeters and accuracy to one decimal place (0.1 cm). Body fat measures were conducted using the TANITA MC-780. We utilized the growth curves established for the Greek population to classify children/adolescents as having obesity, overweight, or normal body mass index (BMI) [19]. Measurements of parents’ height, weight, and BMI were also measured and recorded.

Waist circumference (WC) was measured horizontally at the midpoint between the lower rib and the iliac crest, while hip circumference (HC) was measured at the widest point of the hips and buttocks. Both measurements were taken with the participant standing upright and the measuring tape parallel to the floor. Blood samples were collected at 8:00 a.m. following a 12-h fast, both at the beginning and end of the study (i.e., one year after the intervention), for analysis of hematologic, biochemical, and endocrinologic parameters.

All participants entered a comprehensive, personalized, multidisciplinary, lifestyle intervention program focusing on healthy diet, good quality sleep, and regular physical activity. The lifestyle modification program was individually provided to each child and their family by a team of specialists, including a pediatrician, pediatric endocrinologist, pediatric dietician, professional fitness personal trainer, and, when necessary, a pediatric clinical psychologist. The methodology of this intervention program has also been detailed in previous studies conducted by our team [20,21].

More precisely, during the initial assessment, the pediatric dietician conducted a nutritional evaluation, including a 24-h dietary recall. This involved the documentation of the frequency of meals and snacks, the consumption of sweets and beverages, the methods of meal preparation, the eating habits, and the family meal dynamics. Subsequently, with the assistance of the dietician, the participants completed the “Toybox” food frequency questionnaire. This questionnaire is a 44-item, semi-quantitative, closed-ended questionnaire that assesses the frequency of consumption of specific foods. It includes 37 questions related to the frequency and average consumption of particular food groups, and seven questions about the frequency and consumption of intermediate meals and intake of food supplements. The questionnaire is based on an existing tool designed by Huybrechts [22]. Its final form, along with its validity and reliability testing, was developed as part of the multicentre “ToyBox” study. This study aimed to develop, implement, and evaluate the effectiveness of an intervention for preschool children, with family involvement, across six European countries (Belgium, Bulgaria, Greece, Germany, Poland, and Spain). It focused on four daily life behaviors: snack and water consumption, physical activity, and sedentary behavior. Initially written in English, the questionnaire was translated into local languages and then was re-translated between December 2011 and January 2012. Two members from each participating center, under the constant supervision of the principal researcher, checked the translation process. The final version was confirmed prior to the questionnaires being printed and distributed [23].

The dietician educated the participants and their parents/guardians about childhood obesity, including its widespread prevalence, causes, and long-term adverse effects in adulthood. The goal was to encourage the whole family to combat childhood obesity and embrace a healthier lifestyle [20,24]. Thereafter, the dietician provided guidance on healthier food choices, portion control, regular meal patterns, and adherence to the USDA’s “My Plate” approach [25]. In addition, they discussed the benefits of breakfast for weight management and cognitive function [26], emphasizing the importance of family involvement and reducing the consumption of fast food and sugary beverages. At subsequent assessments, dietary goals were reviewed, and patients were encouraged to maintain adherence while addressing any challenges.

A professional fitness personal trainer assessed the participants’ physical activity levels, recorded the frequency, duration, and intensity of activities, as well as their preferences for various sports. Based on this assessment, personalized physical activity plans were developed to promote enjoyment and adherence. The intervention was intended to recommend a personalized physical activity plan that would be seen not as obligatory, boring, or challenging, but as a highly enjoyable experience. Recommendations included both organized and non-organized activities. Participants were encouraged to engage in another physical activity of their choice, such as walking, jogging, dancing, or cycling, for 30–45 min on the remaining days of the week. Families were advised to minimize sedentary behavior and limit time spent in front of screens, including television, smartphones, and tablets. They were also encouraged to incorporate more physical activity into their daily routines, such as taking the stairs instead of the elevator, or walking and cycling instead of driving. The assessment of the professional personal trainer was performed at each of the follow-up visits, and all information about physical activity and the progress made was recorded.

If needed, participants were referred to a pediatric clinical psychologist, who assessed the family dynamics and offered psychological support to both the children and their parents/guardians. In cases where more severe psychological issues were identified, patients were referred to a mental health service.

In addition, participants were requested to utilize the ‘myBigO’ application (app) for a duration of four weeks before and after the intervention. Specifically, they were instructed to photograph the food items they consumed and any food advertisements encountered in their daily environment. Moreover, they were asked to wear a smartwatch connected to the app for as many hours as possible throughout the day, including at least two school days and one weekend day. They were also encouraged to wear the smartwatch during sleep for a minimum of three nights, including at least two school nights and one weekend night.

More specifically, the smartphone functioned as the central hub, connecting to the smartwatch via Bluetooth to gather data. This setup collected information on physical activity, sleep habits, and GPS location, and enabled participants to capture photos of their meals or food advertisements. The smartwatch provided accelerometer signals and GPS data, which were transmitted to the smartphone. All data were collected in the ‘myBigO’ app and then were transmitted to BigO servers so that individual and aggregated behavior and environment indicators could be extracted [17,18].

All participants were followed-up for one year. The frequency of follow-up examinations varied based on BMI classification. Throughout the lifestyle intervention period, subjects with obesity were followed-up every month, and those with overweight every two months. These reassessments included evaluation by the pediatrician, pediatric endocrinologist, pediatric dietician, and professional fitness personal trainer.

### 2.3. Statistical Analysis

Initially, data were entered into an Excel spreadsheet and subsequently transferred to a corresponding database in SPSS (IBM). Categorical data were presented as absolute frequencies and percentages (%), while continuous data were described using mean and standard deviation (SD) for normally distributed variables. The normality of quantitative variables was assessed through graphical methods, such as histograms, QQ-plots, and the Kolmogorov–Smirnov test. The Mann–Whitney U test was employed to explore correlations between skewed quantitative factors and BMI categories and gender. The T-test was utilized for normally distributed quantitative factors and BMI categories. Chi-square was used for testing correlations between qualitative variables. If conditions for the Chi-square criterion were not met, Fisher’s exact test or Chi-square independence was applied. The z-test of proportions determined statistically significant differences between groups. Variables were assessed at baseline and after 12 months using the Paired T-Test for normally distributed data and the Wilcoxon Rank Test for skewed variables, considering dependent samples. For categorical variables, McNemar’s Bowker Test was used for comparison. All statistical analyses were conducted using SPSS v.25. A significance level of 5% was considered statistically significant for all analyses.

### 2.4. Ethical Isssues

Our study was approved by the Committee on the Ethics of Human Research at “Aghia Sophia” Children’s Hospital. Written informed consent was obtained from a parent or legal guardian in all cases, and assent was given by all participants.

## 3. Results

### 3.1. Demographic and Clinical Characteristics of the Participants at Initial and Annual Assessment

Table 1 illustrates the demographic and clinical characteristics of all participants before and after the intervention. Following the intervention, the study sample consisted of 386 children and adolescents aged 8–18 years (mean age ± standard deviation (SD): 12.495 ± 1.988 years). Among them, 293 (75.9%) were classified as having obesity and 93 (24.1%) as having overweight. There was no statistically significant difference in the anthropometric characteristics (weight, height, BMI) of participants between the two time points.

### 3.2. Descriptive Data of the Participants by Weight Category at Initial and Annual Assessment

The tables below provide the data obtained and evaluated from the questionnaires completed by participants before and after the intervention.

More specifically, Table 2 presents data regarding the frequency of food consumption by weight category. Following the lifestyle intervention, there was a significant decrease in water consumption among subjects with obesity (798.38 ± 266.52 mL vs. 773.60 ± 292.23 mL) and overweight (844.02 ± 241.32 mL vs. 751.14 ± 290.66 mL) (*p* = 0.018). Moreover, at the annual assessment, subjects with obesity demonstrated increased intake of smoothies (169.33 ± 133.46 mL vs. 181.90 ± 143.89 mL, *p* = 0.018), as well as increased consumption of white milk (283.64 ± 152.08 mL, *p* =0.013) compared to subjects with overweight.

Similar results were observed in the consumption of fruit yogurt with flavorings or sugar, indicating that following the intervention, subjects with obesity consumed higher quantities compared to those with overweight (obesity: 111.92 ± 40.53 g vs. overweight: 94.08 ± 44.57 g, *p* = 0.008). However, both groups exhibited decreased consumption compared to pre-intervention levels, although this difference was not statistically significant (*p* = 0.232). Regarding cheese consumption, the amount consumed by subjects in both BMI groups was significantly lower after the intervention compared to that consumed before the intervention (obesity: 30.54 ± 12.89 g vs. 28.52 ± 12.93 g; overweight: 27.82 ± 12.36 g vs. 26.35 ± 12.35 g, *p* = 0.044).

Regarding chocolate intake, at the annual assessment, children and adolescents with obesity consumed higher amounts compared to those with overweight (obesity: 68.68 ± 28.53 g vs. overweight: 60.94 ± 28.50 g, *p* = 0.033). A similar trend was noticed with cake consumption (obesity: 78.99 ± 41.04 g vs. overweight: 67.08 ± 34.75 g, *p* = 0.008), biscuits (obesity: 31.13 ± 14.16 g vs. overweight: 27.40 ± 12.96 g, *p* = 0.034), and bakery products (obesity: 93.25 ± 49.20 g vs. overweight: 77.72 ± 40.94 g, *p* = 0.013). However, in both BMI categories, there was a reduction in the consumption of these products after the intervention, although these differences did not reach statistical significance.

At the annual assessment, all subjects demonstrated a significant reduction in the intake of breakfast cereal with added sugar compared with their intake prior to the intervention (obesity: 31.80 ± 9.72 g vs. 30.93 ± 10.45 g; overweight: 32.95 ± 10.18 g vs. 29.71 ± 12.06 g) (*p* = 0.023).

In terms of white and wholemeal bread consumption, at the annual assessment, children and adolescents with obesity consumed greater quantities compared to those with overweight (*p* = 0.007 and *p* = 0.004, respectively).

**Figure 2:**
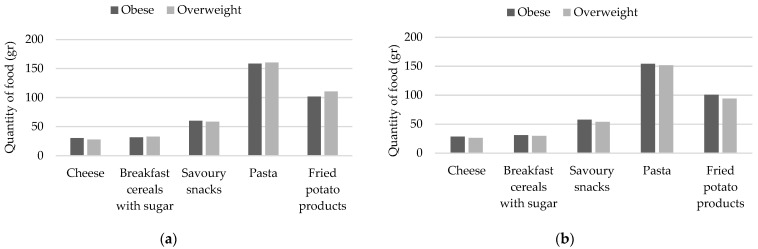
Consumption of certain food quantities by BMI category: (**a**) consumption of certain food quantities by BMI category before the intervention; (**b**) consumption of certain food quantities by BMI category after the intervention.

Finally, both subjects with obesity and overweight demonstrated a significant reduction in the consumption of savory snacks, pasta, and fried potatoes following the intervention, compared to the initial assessment (*p* = 0.020, *p* = 0.014 and *p* = 0.013, respectively). The differences in the quantities of certain foods between the two BMI categories are illustrated in Figure 2a for the initial assessment and Figure 2b for the annual assessment.

Table 3 presents the data on food consumption between meals according to BMI before and after the intervention. Following the intervention, a significant increase in the daily consumption of water between meals was noted in both BMI categories (*p* = 0.001).

Table 4 displays data regarding TV viewing habits during meals. Following the intervention, a significantly greater number of children and adolescents with obesity stopped watching TV during the afternoon meal (*p* = 0.038) and during dinner (*p* = 0.002).

Table 5 presents the distribution of physical activity by BMI category before and after the intervention. At baseline, children and adolescents with overweight spent more time engaged in active play on weekends compared to their peers with obesity (obesity: 131.84 ± 127.90 min/week vs. overweight: 169.95 ± 143.52 min/week, *p* = 0.017). There was no significant difference in the number of participants in both BMI categories who were members of a sports club at the beginning and the end of the study. Similarly, no difference was noted in the time spent participating in sports club activities. In addition, children and adolescents with obesity showed an increase in the time spent in active play on both weekdays (65.62 ± 83.24 min/week vs. 71.62 ± 98.32 min/week) and weekends (131.84 ± 127.90 min/week vs. 136.15 ± 134.97 min/week) after the intervention, however, these results did not reach statistical significance (*p* = 0.443 and *p* = 0.870, accordingly).

### 3.3. Descriptive Data of the Participants by Gender at Initial and Annual Assessment

**Figure 3:**
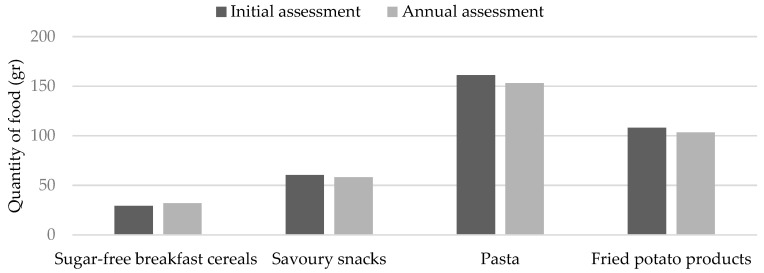
Consumption of certain food quantities among boys before and after the intervention.

Table 6 presents data on food consumption frequency categorized by gender at the initial assessment and annual assessment. Initially, boys consumed greater amounts of water (boys: 825.13 ± 261.66 mL vs. girls: 792.24 ± 259.79 mL, *p* = 0.031), cheese (boys: 31.25 ± 13.31 g vs. girls: 28.39 ± 12.09 g, *p* = 0.049), meat products (boys: 29.91 ± 13.13 gr vs. girls: 26.75 ± 11.62 g, *p* = 0.018), fried potato products (boys: 108.02 ± 43.34 g vs. girls: 100.00 ± 32.70 g, *p* = 0.028), and potatoes (boys: 108.23 ± 43.43 g vs. girls: 94.61 ± 41.45 g, *p* = 0.002). Following the intervention, boys consumed higher amounts of fried potatoes (boys: 103.33 ± 31.30 g vs. girls: 94.87 ± 26.14 g, *p* = 0.010), less water (825.13 ± 261.66 mL vs. 762.76 ± 299.05 mL, *p* = 0.015), more sugar-free breakfast cereals (29.30 ± 9.54 g vs. 31.92 ± 9.89 g, *p* = 0.031), fewer savory snacks (60.31 ± 16.44 g vs. 55.65 ± 18.58 g, *p* = 0.002), less pasta (161.13 ± 39.02 g vs. 153.21 ± 39.53 g, *p* = 0.023), and fewer fried potato products (108.02 ± 32.45 g vs. 103.33 ± 31.30 gr, *p* = 0.050). In contrast, girls consumed more smoothies (172.22 ± 129.80 mL vs. 188.46 ± 141.63 mL, *p* = 0.023) and less cooked vegetables (120.10 ± 68.31 g vs. 112.55 ± 69.13 g, *p* = 0.028) after the intervention compared to the initial assessment. The differences in the quantities of certain foods among boys are illustrated in Figure 3 for the initial assessment and annual assessment.

Table 7 presents data on food consumption between meals categorized by gender before and after the intervention. Following the intervention, a greater number of boys consumed water between meals daily (initial assessment: 71 vs. annual assessment: 90, *p ≤* 0.001).

Table 8 presents the data on physical activity according to gender before and after the intervention.

## 4. Discussion

In our study, we determined the dietary and physical activity habits of children and adolescents with overweight and obesity before and after the implementation of a comprehensive, personalized, lifestyle intervention program using objective data collection through the ‘myBigO’ application. It is important to emphasize that this study was conducted during the Covid-19 lockdown period, which should be taken into consideration when interpreting the results. We demonstrated that, following the lifestyle intervention for one year, 293 participants (75.9%) had obesity and 93 (24.1%) had overweight. Meanwhile, there was no statistically significant difference in the anthropometric parameters of the participants compared to their measurements at initial assessment.

These findings concur with some meta-analyses [27,28] and studies of e-Health interventions [29], which showed no improvement in participants’ anthropometric indicators after the intervention. This result, therefore, is not considered unexpected as it has been shown that e-Health interventions targeting childhood obesity can be effective even if they achieve maintenance and no further increase in participants’ body weight and BMI [30,31]. Moreover, using only anthropometric indicators as the primary outcome in intervention studies targeting childhood obesity is inadequate for assessing the intervention’s impact [28]. It has been shown that e-Health interventions in children and adolescents can improve anthropometric data and lead to weight loss by promoting healthy lifestyle habits [30]. Therefore, although no change in anthropometric measurements was observed in our study, significant improvements in participants’ dietary and exercise habits were evident. These habits have been extensively studied for their positive or negative impact on childhood and adolescent obesity, aligning with the results of the majority of the above studies [14,27,28,29,30,31,32,33,34].

Regarding the dietary habits, a significant finding was that, following the intervention, both participants with obesity and overweight demonstrated a significant decrease in the consumption of cheese, breakfast cereal with added sugar, savory snacks, pasta, and fried potatoes. In children and adolescents, larger portion sizes of food are associated with higher energy intake and weight gain [35,36]. Furthermore, a recent study found a positive correlation between portion size of certain energy-dense foods, such as cheese and breakfast cereal, and BMI in adolescents [37].

More specifically, the evidence regarding cheese consumption and its relationship to childhood obesity is controversial. Some studies have found no association [38,39]. Indeed, a systematic review suggests potential beneficial effects of dairy consumption in general on childhood and adolescent overweight and obesity due to components, such as protein, calcium, and bioactive peptides [39]. On the other hand, other studies conducted in both children and adults have found a positive association between cheese consumption and obesity [40,41,42,43]. This positive association may be attributed to the high energy density and increased fat content, particularly saturated fat, found in cheese [42,44,45]. In addition, the high sodium content in cheese may contribute to the increased consumption of sugary, calorie-dense drinks, thereby promoting weight gain [44].

Regarding the consumption of breakfast cereal with added sugar, several studies demonstrate a negative correlation between breakfast cereal consumption and obesity in children and adolescents, regardless of its sugar content [46,47,48,49,50], or that there is no difference between different types of breakfast cereal (sugary or unsweetened) and participants’ anthropometric measurements [49]. However, breakfast cereals have also been associated positively with BMI in children and adolescents due to ingredients like sugars, nuts, honey, and fruit, which increase its energy density [37]. A recent study conducted in children and adolescents in Greece revealed that the increased sugar intake increases the risk of overweight and obesity, with processed breakfast cereal being the second major source of simple sugar intake in the participants’ diets [51].

Regarding the consumption of savory snacks and obesity in children and adolescents, the majority of the literature indicates a positive correlation [52,53,54]. Specifically, a study involving 8–10-year-old children revealed that, with each weekly portion of salty snacks consumed, the likelihood of obesity increased by 2% [52]. The mechanisms underlying this association include the elevated energy density of salty snacks, which contributes additional calories to daily energy intake and promotes a positive energy balance [55,56]. Another contributing factor is the increased sodium content in these snacks, which has been linked to increased rates of overweight and obesity in children [54,57]. Furthermore, studies suggest that high salt intake may lead to increased consumption of sugary soft drinks, further contributing to weight gain [44,58].

Concerning pasta consumption, the evidence is conflicting. One study involving children suggested that surpassing the recommended pasta intake may contribute to an increased BMI [59]. This positive association could be attributed to the fact that pasta is usually paired with other high-saturated fat foods, such as meat and cheese [60]. At the same time, an adult study found either an inverse relationship or no association between pasta consumption and body weight when consumed as a part of a balanced diet. The existence of an inverse relationship could be attributed to slower carbohydrate absorption and a reduced glycemic response following pasta consumption [60]. However, the mechanisms underlying both the positive and inverse associations remain unclear. Therefore, in our study, the benefit of reducing the amount of pasta consumed post-intervention can be attributed to the fact that the portion size and subsequently the caloric intake was reduced leading to a negative energy balance and improvement in body weight.

Regarding the consumption of fried potatoes, the majority of the literature indicates a positive association with overweight and obesity in children and adolescents [61,62,63,64]. The primary mechanism behind this association is the increased caloric content of these products, which can range from 383 to 574 calories per 100 g. Furthermore, chips, in particular, possess a low satiety index, prompting individuals to consume large quantities until feeling satiated [62].

From the above evidence, it can be concluded that the observed reduction in the amount of these foods following our intervention may positively impact the management of childhood and adolescent obesity.

Further to the above, following our intervention, there was an increase in the number of participants across both BMI categories, who consumed water between meals on a daily basis. The existing literature recommends substituting sugary soft drinks with water during meals for the management of childhood obesity, as it reduces energy intake and the caloric content of the meal [65,66,67]. Furthermore, a meta-analysis involving clinical trials in adults showed that consuming water before and after meals can lead to weight reduction. Possible mechanisms that explain the water’s contribution to weight management include increased thermogenesis, reduced hunger, increased satiety resulting in lower energy intake, and the substitution of sugary drinks [68]. Based on the above, the finding from our study can be viewed as positive for the management of childhood and adolescent obesity.

Finally, another finding of our study is that, following the implementation of lifestyle intervention for one year, fewer children and adolescents with obesity watched TV during the evening snack and dinner. This finding can be considered positive, as the adverse impact of screen time on overweight and obesity in children and adolescents has been widely documented [69,70]. The primary mechanisms underlying this relationship include increased food intake and the consumption of processed foods, which are typically high in sugars, fats, and salt, while engaging with screens [71,72,73]. Exposure to food advertisements, particularly those promoting unhealthy foods, is another mechanism linking increased screen time to childhood overweight and obesity [74,75,76], as well as decreased physical activity and inadequate sleep resulting from screen viewing [69,77].

The strengths of our study include the objective recording and assessment of participants’ dietary and exercise habits through the utilization of the ‘myBigO’ app of the BigO program. The results of our study were not solely based on self-administered questionnaires. Indeed, the ‘myBigO’ app was well received by the participants. Furthermore, each participant received personalized advice on lifestyle interventions by a multidisciplinary team with expertise on the prevention and management of obesity. In addition, the strengths of the study may include the large sample size, as well as the long follow-up period of the participants.

The main limitation of our study arises from the constraints imposed by the COVID-19 pandemic, which imposed greater difficulties in adhering to the diet, sleep, and exercise program. This restriction complicated our contact with certain participants, leading to the absence of data from their follow-ups. As a result, they could not be included in our final sample. Another limitation of our study is the absence of a control group. Recruiting a comparable number of subjects with normal BMI who would not receive an intervention was not feasible, as our sample was drawn from the Center for the Prevention and Management of Overweight and Obesity in Childhood and Adolescence. Participants were referred by their pediatrician or general practitioner, with the goal of reducing their BMI, and they were all motivated and willing to take part in our study. Furthermore, the aim of our study was to determine the dietary and physical activity habits of children and adolescents with overweight and obesity before and after the implementation of a lifestyle intervention program for one year. Therefore, the subjects served as their own control.

## 5. Conclusions

In conclusion, obesity in childhood and adolescence is a major public health problem with many significant health and financial implications. Many risk factors, including diet and exercise habits, trigger the development of the disease. Today, there is a need for e-Health interventions to change diet and exercise habits, and prevent and treat childhood and adolescent obesity.

In our study, we determined objectively (through the ‘myBigO’ app) the diet and exercise habits of children and adolescents with overweight and obesity before and after the implementation of a comprehensive, multidisciplinary, personalized, lifestyle intervention program within the context of the HORIZON Program ‘BigO: Big Data against Childhood Obesity’. Following the implementation of this program for one year, significant improvements in the participants’ diet and exercise habits were observed, despite the Covid-19 pandemic. We anticipate that these positive results will contribute to the development of health policies aiming to prevent and manage the epidemic of childhood obesity.

## Figures and Tables

**Table 1:** Demographic and clinical characteristics of all participants before and after the intervention.

	Initial Assessment			Annual Assessment			
	Obesity	Overweight		Obesity	Overweight		
	N (%) 290 (75.1%)	N (%) 96 (24.9%)	*p*-Value	N (%) 293 (75.9%)	N (%) 93 (24.1%)	*p*-Value	*p*_between time points_
Gender							
Males	155 (77.9%)	44 (22.1%)		153 (76.9%)	46 (23.1%)		
Females	135 (72.2%)	52 (27.8%)	0.196	140 (74.9%)	47 (25.1%)	0.643	**-**
Age, years	12.338 ± 2.036	12.969 ± 1.759	**0.004**	13.554 ± 1.901	13.307 ± 2.239	0.158	**<0.001**
Height, cm	156.662 ± 12.912	159.150 ± 12.057	0.097	158.855 ± 12.360	158.668 ± 11.492	0.933	0.079
Weight, kg	73.957 ± 20.792	62.509 ± 13.104	**<0.001**	74.962 ± 19.470	62.090 ± 12.741	**<0.001**	0.318
BMI, kg/m^2^	29.534 ± 4.744	24.366 ± 2.169	**<0.001**	29.199 ± 4.173	24.348 ± 2.039	**<0.001**	0.824

Abbreviations: BMI, body mass index; continuous variables are presented as mean ± standard deviation (SD) and categorical variables as frequencies (percentages); significance values are derived from comparisons between the two categories of BMI using the Mann–Whitney U test for variables that do not follow a normal distribution, T-test for variables that are normally distributed, and Pearson’s X^2^ test for categorical variables; *p*-values between time points were obtained from comparisons between the two assessments using the Paired T-test or Wilcoxon’s signed rank test for variables that do not follow a normal distribution; statistically significant correlations are shown in bold.

**Table 2:** Frequency of food consumption by BMI category before and after the intervention.

	Initial Assessment			Annual Assessment			
	Obesity	Overweight		Obesity	Overweight		
	N (%) 290 (75.1%)	N (%) 96 (24.9%)	*p*-Value	N (%) 293 (75.9%)	N (%) 93 (24.1%)	*p*-Value	*p*_between time points_
Water (mL)	798.38 ± 266.52	844.02 ± 241.32	0.091	773. 60 ± 292.23	751.14 ± 290.66	0.476	**0.018**
Beverages/soft drinks with added sugar (mL)	244.83 ± 149.95	255.00 ± 134.92	0.352	241.12 ± 156.38	216.18 ± 142.06	0.219	0.173
Soft drinks/light refreshments (mL)	201.55 ± 156.13	204.10 ± 160.80	0.988	228.47 ± 163.98	227.50 ± 190.54	0.599	0.068
Fruit juice, homemade, freshly squeezed (mL)	253.24 ± 153.44	263.41 ± 134.96	0.235	266.09 ± 139.68	241.03 ± 120.80	0.224	0.242
Fruit juice, packaged, bottled (mL)	236.51 ± 147.46	237.01 ± 140.82	0.842	236.81 ± 138.20	203.03 ± 102.61	0.059	0.467
Tea (mL)	188.50 ± 125.78	183.80 ± 126.43	0.785	194.61 ± 127.58	183.85 ± 103.50	0.751	0.397
Smoothies (mL)	169.33 ± 133.46	188.78 ± 144.07	0.437	181.90 ± 143.89	180.77 ± 130.67	0.832	**0.018**
Milk, white (mL)	278.92 ± 168.03	286.36 ± 139.94	0.264	283.64 ± 152.08	237.36 ± 103.21	**0.013**	0.635
Sweetened or chocolate milk (mL)	190.37 ± 147.22	159.80 ± 141.78	0.134	180.06 ± 127.22	147.78 ± 96.50	0.199	0.306
Plain yoghurt (g)	129.09 ± 40.47	131.83 ± 43.91	0.629	131.37 ± 42.50	124.66 ± 42.99	0.239	0.932
Fruit yoghurt or yoghurt with flavouring or sugar (g)	114.71 ± 39.29	107.06 ± 42.77	0.192	111.92 ± 40.53	94.08 ± 44.57	**0.008**	0.232
Cheese (g)	30.54 ± 12.89	27.82 ± 12.36	0.064	28.52 ± 12.93	26.35 ± 12.35	0.165	**0.044**
Canned fruit (g)	43.75 ± 17.83	39.50 ± 10.69	0.358	43.90 ± 21.80	40.15 ± 13.30	0.429	0.244
Fresh fruit (g)	155.49 ± 67.66	162.39 ± 73.44	0.485	159.35 ± 66.77	156.82 ± 65.87	0.701	0.355
Raw vegetables (g)	139.34 ± 64.09	133.59 ± 70.49	0.416	137.45 ± 67.04	141.63 ± 71.50	0.835	0.295
Cooked vegetables (g)	124.15 ± 69.07	123.93 ± 68.25	0.986	119.55 ± 70.21	118.17 ± 67.68	0.953	0.136
Chocolate (g)	69.62 ± 28.19	69.07 ± 30.13	0.815	68.68 ± 28.54	60.94 ± 28.50	**0.033**	0.436
Milk-based desserts (g)	78.68 ± 37.38	74.40 ± 32.43	0.829	76.97 ± 37.88	71.79 ± 36.36	0.225	0.574
Cake (g)	83.34 ± 47.50	73.67 ± 37.71	0.207	78.99 ± 41.04	67.08 ± 34.75	**0.008**	0.444
Biscuits (g)	32.26 ± 15.28	31.60 ± 14.66	0.913	31.13 ± 14.16	27.40 ± 12.96	**0.034**	0.322
Baked goods (g)	91.43 ± 42.07	90.10 ± 47.74	0.472	93.25 ± 49.20	77.72 ± 40.94	**0.013**	0.917
Desserts with sugar (g)	11.87 ± 8.38	11.89 ± 7.91	0.790	11.26 ± 7.60	10.31 ± 7.31	0.522	0.101
Sugar-free breakfast cereals (g)	29.51 ± 9.71	30.00 ± 10.13	0.742	30.76 ± 10.11	31.94 ± 10.69	0.415	0.069
Breakfast cereals with sugar (g)	31.80 ± 9.72	32.95 ± 10.18	0.402	30.93 ± 10.45	29.71 ± 12.06	0.495	**0.023**
White bread (g)	68.82 ± 34.73	72.00 ± 38.81	0.743	68.86 ± 34.74	57.78 ± 29.63	**0.007**	0.059
Black or wholemeal bread (g)	52.91 ± 23.66	58.24 ± 29.72	0.531	53.49 ± 24.66	45.61 ± 20.54	**0.004**	0.509
Savoury snacks (g)	60.26 ± 17.63	58.68 ± 16.34	0.339	57.73 ± 18.27	53.99 ± 18.99	0.143	**0.020**
Meat and poultry (g)	108.10 ± 22.99	106.39 ± 27.40	0.932	107.48 ± 25.52	104.78 ± 26.35	0.395	0.851
Fish and seafood (g)	99.15 ± 28.90	101.08 ± 30.25	0.400	98.73 ± 31.74	99.11 ± 28.77	0.511	0.448
Meat products (g)	27.97 ± 12.19	29.61 ± 13.43	0.461	27.78 ± 13.00	25.90 ± 12.41	0.214	0.166
Pasta (g)	158.46 ± 41.37	160.23 ± 39.69	0.746	154.03 ± 40.28	151.72 ± 37.89	0.626	**0.014**
Rice (g)	140.30 ± 40.82	149.10 ± 39.71	0.085	138.53 ± 39.14	137.07 ± 32.77	0.847	0.058
Fried potato products (g)	101.85 ± 33.51	110.76 ± 29.60	**0.040**	100.82 ± 28.62	94.08 ± 30.51	0.078	**0.013**
Potatoes (g)	100.74 ± 42.18	104.57 ± 45.33	0.586	103.18 ± 42.58	101.60 ± 41.94	0.758	0.399
Chocolate spread (g)	19.98 ± 10.63	19.47 ± 10.74	0.547	19.58 ± 10.97	17.92 ± 9.22	0.339	0.491
Legumes (g)	117.59 ± 47.13	119.83 ± 46.85	0.678	118.51 ± 44.36	117.81 ± 47.89	0.981	0.621

Continuous variables are presented as mean ± standard deviation; significance values are derived from comparisons between the two categories of BMI using the Mann–Whitney U test for variables that do not follow a normal distribution, *t*-test for variables that are normally distributed, *p*-values between time points were obtained from comparisons between the two assessments using Wilcoxon’s signed rank test for variables that do not follow a normal distribution; statistically significant correlations are shown in bold.

**Table 3:** Distribution of food consumption between meals by BMI category before and after intervention.

	Initial Assessment			Annual Assessment			
	Obesity	Overweight		Obesity	Overweight		
	N (%)	N (%)		N (%)	N (%)		
	290 (75.1%)	96 (24.9%)	*p*-Value	293 (75.9%)	93 (24.1%)	*p*-Value	*p*_between time points_
How often does the child eat between meals *:							
(Almost) never	18 (6.4%)	5 (5.4%)		23 (8.2%)	11 (12.1%)		
1–3 days/month	16 (5.7%)	8 (8.7%)		21 (7.4%)	9 (9.9%)		
1 day/week	23 (8.2%)	6 (6.5%)		28 (9.9%)	7 (7.7%)		
2–4 days/week	60 (21.4%)	25 (27.2%)		71 (25.2%)	18 (19.8%)		
5–6 days/week	32 (11.4%)	9 (9.8%)		31 (11.0%)	8 (8.8%)		
Daily	132 (47.0%)	39 (42.4%)	0.726	108 (38.3%)	38 (41.8%)	0.636	0.374
How often does the child drink between meals *:							
(Almost) never	34 (12.3%)	13 (14.3%)		32 (11.5%)	17 (19.5%)		
1–3 days/month	23 (8.3%)	7 (7.7%)		24 (8.6%)	7 (8.0%)		
1 day/week	49 (17.8%)	11 (12.1%)		22 (7.9%)	3 (3.4%)		
2–4 days/week	51 (18.5%)	15 (16.5%)		51 (18.3%)	15 (17.2%)		
5–6 days/week	19 (6.9%)	9 (9.9%)		17 (6.1%)	6 (6.9%)		
Daily	100 (36.2%)	36 (39.6%)	0.738	132 (47.5%)	39 (44.8%)	0.375	**0.001**

*: except breakfast, lunch, dinner. Variables are presented as frequencies (percentages); significance values are derived from comparisons between the two categories of BMI using the Pearson’s X^2^ test; *p*-values between time points were derived from comparisons between the two assessments using McNemar’s Bowker Test for categorical variables; statistically significant correlations are shown in bold.

**Table 4:** Distribution of TV watching during meals by BMI category before and after intervention.

	Initial Assessment			Annual Assessment			
	Obesity	Overweight		Obesity	Overweight		
	N (%)	N (%)		N (%)	N (%)		
	290 (75.1%)	96 (24.9%)	*p*-Value	293 (75.9%)	93 (24.1%)	*p*-Value	*p*_between time points_
1. Breakfast							
Never	197 (67.9%)	72 (75.0%)		193 (65.9%)	62 (66.7%)		
Rarely	36 (12.4%)	7 (7.3%)		36 (12.3%)	12 (12.9%)		
Sometimes	20 (6.9%)	7 (7.3%)		20 (6.8%)	10 (10.8%)		
Often	17 (5.9%)	6 (6.3%)		22 (7.5%)	5 (5.4%)		
Always	20 (6.9%)	4 (4.2%)	0.539	22 (7.5%)	4 (4.3%)	0.564	0.291
2. Morning snack							
Never	223 (76.9%)	70 (72.9%)		223 (76.1%)	66 (71.0%)		
Rarely	40 (13.8%)	12 (12.5%)		31 (10.6%)	17 (18.3%)		
Sometimes	14 (4.8%)	8 (8.3%)		17 (5.8%)	5 (5.4%)		
Often	8 (2.8%)	3 (3.1%)		12 (4.1%)	2 (2.2%)		
Always	5 (1.7%)	3 (3.1%)	0.642	10 (3.4%)	3 (3.2%)	0.357	0.653
3. Lunch							
Never	96 (33.1%)	31 (32.3%)		103 (35.2%)	31 (33.3%)		
Rarely	49 (16.9%)	10 (10.4%)		41 (14.0%)	15 (16.1%)		
Sometimes	62 (21.4%)	33 (34.4%)		65 (22.2%)	27 (29.0%)		
Often	49 (16.9%)	13 (13.5%)		45 (15.4%)	12 (12.9%)		
Always	34 (11.7%)	9 (9.4%)	0.099	39 (13.3%)	8 (8.6%)	0.517	0.336
4. Afternoon meal							
Never	116 (40.0%)	45 (46.9%)		133 (45.4%)	46 (49.5%)		
Rarely	53 (18.3%)	10 (10.4%)		45 (15.4%)	14 (15.1%)		
Sometimes	52 (17.9%)	21 (21.9%)		52 (17.7%)	19 (20.4%)		
Often	52 (17.9%)	15 (15.6%)		40 (13.7%)	9 (9.7%)		
Always	17 (5.9%)	5 (5.2%)	0.353	23 (7.8%)	5 (5.4%)	0.735	**0.038**
5. Dinner							
Never	93 (32.1%)	28 (29.2%)		83 (28.3%)	29 (31.2%)		
Rarely	33 (11.4%)	12 (12.5%)		37 (12.6%)	6 (6.5%)		
Sometimes	50 (17.2%)	18 (18.8%)		62 (21.2%)	31 (33.3%)		
Often	77 (26.6%)	29 (30.2%)		59 (20.1%)	19 (20.4%)		
Always	37 (12.8%)	9 (9.4%)	0.843	52 (17.7%)	8 (8.6%)	**0.029**	**0.002**
6. Snack before bedtime							
Never	104 (36.0%)	32 (33.3%)		103 (35.2%)	37 (39.8%)		
Rarely	25 (8.7%)	9 (9.4%)		31 (10.6%)	5 (5.4%)		
Sometimes	50 (17.3%)	18 (18.8%)		47 (16.0%)	16 (17.2%)		
Often	67 (23.2%)	23 (24.0%)		65 (22.2%)	25 (26.9%)		
Always	43 (14.9%)	14 (14.6%)	0.990	47 (16.0%)	10 (10.8%)	0.339	0.129

Variables are presented as frequencies (percentages), significance values are derived from comparisons between the two categories of BMI using the Pearson’s X^2^ test, *p*-values between time points were derived from comparisons between the two assessments using McNemar’s Bowker Test for categorical variables, and statistically significant correlations are shown in bold.

**Table 5:** Distribution of physical activity by BMI category before and after the intervention.

	Initial Assessment			Annual Assessment			
	Obesity	Overweight		Obesity	Overweight		
	N (%)	N (%)	*p*-Value	N (%)	N (%)	*p*-Value	*p*_between time points_
	290 (75.1%)	96 (24.9%)		293 (75.9%)	93 (24.1%)		
Is the child a member of a sports club?							
Yes	184 (63.4%)	58 (60.4%)		175 (59.7%)	57 (61.3%)		
No	106 (36.6%)	38 (39.6%)	0.594	118 (40.3%)	36 (38.7%)	0.789	0.353
Time the child spends in the sports club (minutes/week)	166.03 ± 178.02	187.92 ± 210.44	0.540	159.39 ± 171.09	177.69 ± 177.57	0.297	0.414
Time the child spent in active play yesterday (minutes/week)	65.62 ± 83.24	81.88 ± 110.90	0.310	71.62 ± 98.32	84.52 ± 100.11	0.225	0.443
Time the child spent in active play on the most recent weekend (minutes/week)	131.84 ± 127.90	169.95 ± 143.52	**0.017**	136.15 ± 134.97	151.88 ± 142.21	0.342	0.870

Continuous variables are presented as mean ± standard deviation and categorical variables as frequencies (percentages); significance values are derived from comparisons between the two ΒΜΙ categories using the Mann–Whitney U test for variables that do not follow a normal distribution and the Pearson’s X^2^ test for categorical variables, *p*-values between time points are derived from comparisons between the two ratings using Wilcoxon’s signed rank test for variables not following the normal distribution, and McNemar’s Bowker Test for categorical variables; statistically significant correlations are shown in bold.

**Table 6:** Frequency of food consumption by gender before and after the intervention.

	Initial Assessment			Annual Assessment			
	Boys	Girls		Boys	Girls		
	N (%)	N (%)	*p*-Value	N (%)	N (%)	*p*-Value	*p*_between time points_
	199 (51.6%)	187 (48.4%)		199 (51.6%)	187 (48.4%)		
Water (mL)	825.13 ± 261.66	792.94 ± 259.79	**0.031**	762.76 ± 299.05	774.18 ± 284.29	0.904	**0.015**/0.358
Beverages/soft drinks with added sugar (mL)	254.22 ± 160.06	239.73 ± 128.51	0.544	244.62 ± 166.99	226.32 ± 137.98	0.516	0.195/0.556
Soft drinks/light refreshments (mL)	212.96 ± 166.95	190.00 ± 144.59	0.389	232.99 ± 176.62	222.80 ± 162.33	0.679	0.277/0.131
Fruit juice, homemade, freshly squeezed (mL)	254.22 ± 163.71	257.36 ± 132.66	0.471	254.57 ± 131.66	266.46 ± 140.24	0.437	0.650/0.223
Fruit juice, packaged, bottled (mL)	244.21 ± 156.79	228.57 ± 132.82	0.435	237.66 ± 146.56	220.75 ± 114.20	0.452	0.493/0.724
Tea (mL)	190.29 ± 127.24	183.61 ± 124.38	0.691	205.47 ± 130.90	179.79 ± 112.60	0.161	0.208/0.931
Smoothies (mL)	176.00 ± 142.57	172.22 ± 129.80	0.937	175.23 ± 139.78	188.46 ± 141.63	0.468	0.271/**0.023**
Milk, white (mL)	282.89 ± 182.58	278.40 ± 134.60	0.352	265.41 ± 137.69	279.82 ± 148.48	0.245	0.619/0.860
Sweetened or chocolate milk (mL)	197.71 ± 148.20	167.48 ± 143.09	0.112	185.71 ± 132.11	160.38 ± 109.48	0.231	0.207/0.813
Plain yoghurt (gr)	129.13 ± 40.54	130.48 ± 42.23	0.784	128.80 ± 44.35	130.88 ± 40.82	0.666	0.913/0.996
Fruit yoghurt or yoghurt with flavouring or sugar (g)	113.29 ± 40.34	112.27 ± 40.28	0.857	107.02 ± 42.87	109.78 ± 40.93	0.577	0.209/0.735
Cheese (g)	31.25 ± 13.31	28.39 ± 12.09	**0.049**	29.19 ± 12.67	26.76 ± 12.87	0.063	0.130/0.192
Canned fruit (g)	44.72 ± 18.01	40.00 ± 13.85	0.118	45.97 ± 24.88	40.23 ± 13.93	0.277	0.134/0.953
Fresh fruit (g)	161.27 ± 70.64	153.00 ± 67.47	0.324	159.73 ± 64.60	157.67 ± 68.58	0.715	0.925/0.211
Raw vegetables (g)	141.99 ± 62.82	133.55 ± 68.52	0.154	143.74 ± 69.22	132.92 ± 66.58	0.192	0.403/0.517
Cooked vegetables (g)	127.59 ± 69.14	120.10 ± 68.31	0.274	125.37 ± 69.39	112.55 ± 69.13	0.052	0.945/**0.028**
Chocolate (g)	69.00 ± 28.21	70.05 ± 29.13	0.678	67.35 ± 29.27	66.32 ± 28.14	0.808	0.826/0.343
Milk-based desserts (g)	77.86 ± 34.00	77.33 ± 39.03	0.726	75.00 ± 38.02	76.52 ± 37.13	0.502	0.505/0.880
Cake (g)	82.86 ± 44.57	78.92 ± 46.30	0.317	80.35 ± 44.20	72.12 ± 34.68	0.146	0.528/0.654
Biscuits (g)	32.80 ± 15.39	31.37 ± 14.84	0.361	30.92 ± 14.45	29.50 ± 13.41	0.541	0.440/0.536
Baked goods (g)	92.81 ± 43.89	89.08 ± 43.16	0.396	91.07 ± 47.10	87.38 ± 48.58	0.235	0.993/0.881
Desserts with sugar (g)	11.81 ± 8.35	11.95 ± 8.17	0.726	11.80 ± 7.94	10.25 ± 7.03	0.095	0.530/0.104
Sugar-free breakfast cereals (g)	29.30 ± 9.54	30.00 ± 10.11	0.588	31.92 ± 9.89	30.22 ± 10.52	0.191	**0.031**/0.627
Breakfast cereals with sugar (g)	31.90 ± 9.81	32.27 ± 9.89	0.761	31.43 ± 10.97	29.87 ± 10.61	0.255	0.092/0.128
White bread (g)	71.34 ± 35.87	67.69 ± 35.58	0.256	68.24 ± 34.74	63.82 ± 32.80	0.263	0.266/0.127
Black or wholemeal bread (g)	54.54 ± 25.27	53.93 ± 25.56	0.854	54.56 ± 26.63	48.07 ± 20.02	0.128	0.815/0.204
Savoury snacks (g)	60.31 ± 16.44	59.40 ± 18.31	0.863	55.65 ± 18.58	58.21 ± 18.34	0.224	**0.002**/0.937
Meat and poultry (g)	107.58 ± 24.89	107.79 ± 23.35	0.508	105.12 ± 26.07	108.61 ± 25.29	0.056	0.343/0.391
Fish and seafood (g)	100.67 ± 28.46	98.55 ± 29.93	0.450	98.38 ± 30.39	99.31 ± 31.72	0.418	0.568/0.604
Meat products (g)	29.91 ± 13.13	26.75 ± 11.62	**0.018**	28.17 ± 13.67	26.48 ± 11.95	0.371	0.192/0.569
Pasta (g)	161.13 ± 39.02	156.54 ± 42.80	0.325	153.21 ± 39.53	153.77 ± 39.97	0.890	**0.023**/0.244
Rice (g)	144.03 ± 39.64	140.74 ± 41.76	0.434	139.97 ± 37.96	136.16 ± 37.29	0.355	0.430/0.052
Fried potato products (g)	108.02 ± 32.45	100.00 ± 32.70	**0.028**	103.33 ± 31.30	94.87 ± 26.14	**0.010**	**0.050**/0.118
Potatoes (g)	108.23 ± 43.43	94.61 ± 41.45	**0.002**	105.59 ± 41.46	100.00 ± 43.21	0.128	0.687/0.142
Chocolate spread (g)	20.69 ± 10.99	18.93 ± 10.21	0.121	19.89 ± 11.43	18.52 ± 9.70	0.743	0.846/0.382
Legumes (g)	118.67 ± 46.45	117.59 ± 47.73	0.841	117.27 ± 46.76	119.48 ± 43.54	0.706	0.952/0.435

Variables are presented as mean ± standard deviation, and significance values are derived from comparisons between the two gender categories using the Mann–Whitney U test for variables that do not follow the normal distribution; statistically significant correlations are shown in bold.

**Table 7:** Distribution of food consumption between meals by gender before and after the intervention.

	Initial Assessment			Annual Assessment			
	Boys	Girls		Boys	Girls		
	N (%)	N (%)	*p*-Value	N (%)	N (%)	*p*-Value	*p*_between time points_
	199 (51.6%)	187 (48.4%)		199 (51.6%)	187 (48.4%)		
How often does the child eat between meals *:							
(Almost) never	11 (5.8%)	12 (6.6%)		16 (8.3%)	18 (9.9%)		
1–3 days/month	16 (8.4%)	8 (4.4%)		18 (9.4%)	12 (6.6%)		
1 day/week	17 (8.9%)	12 (6.6%)	0.469	21 (10.9%)	14 (7.7%)	0.729	0.808/0.623
2–4 days/week	45 (23.6%)	40 (22.0%)		47 (24.5%)	42 (23.2%)		
5–6 days/week	22 (11.5%)	19 (10.4%)		19 (9.9%)	20 (11.0%)		
Daily	80 (41.9%)	91 (50.0%)		71 (37.0%)	75 (41.4%)		
How often does the child drink between meals *:							
(Almost) never	21 (11.1%)	26 (14.6%)		25 (13.3%)	24 (13.6%)		
1–3 days/month	15 (7.9%)	15 (8.4%)		19 (10.1%)	12 (6.8%)		
1 day/week	32 (16.9%)	28 (15.7%)	0.928	10 (5.3%)	15 (8.5%)	0.723	<**0.001**/0.590
2–4 days/week	34 (18.0%)	32 (18.0%)		32 (17.0%)	34 (19.2%)		
5–6 days/week	16 (8.5%)	12 (6.7%)		12 (6.4%)	11 (6.2%)		
Daily	71 (37.6%)	65 (36.5%)		90 (47.9%)	81 (45.8%)		

*: except breakfast, lunch, dinner. Variables are presented as frequencies (percentages); significance values are derived from comparisons between the two categories of gender using the Pearson’s X^2^ test; *p*-values between time points were derived from comparisons between the two assessments using McNemar’s Bowker Test for categorical variables; statistically significant correlations are shown in bold.

**Table 8:** Distribution of physical activity by gender before and after the intervention.

	Initial Assessment			Annual Assessment			
	Boys	Girls		Boys	Girls		
	N (%)	N (%)	*p*-Value	N (%)	N (%)	*p*-Value	*p*_between time points_
	199 (51.6%)	187 (48.4%)		199 (51.6%)	187 (48.4%)		
Is the child a member of a sports club?							
Yes	126 (63.3%)	116 (62.0%)		119 (59.8%)	113 (60.4%)		
No	73 (36.7%)	71 (38.0%)	0.794	80 (40.2%)	74 (39.6%)	0.900	**<0.001**/**<0.001**
Time the child spends in the sports club (minutes/week)	182.46 ± 192.10	159.79 ± 180.27	0.293	163.24 ± 185.77	164.39 ± 157.91	0.559	0.177/0.817
Time the child spent in active play yesterday (minutes/week)	72.76 ± 89.90	66.36 ± 92.33	0.432	89.47 ± 111.52	59.04 ± 80.48	**0.023**	0.116/0.506
Time the child spent in active play on the most recent weekend (minutes/week)	151.91 ± 145.51	130.05 ± 117.10	0.303	164.01 ± 148.74	114.33 ± 117.75	**0.001**	0.413/0.260

Continuous variables are presented as mean ± standard deviation and categorical variables as frequencies (percentages); significance values are derived from comparisons between the two gender categories using the Mann–Whitney U test for variables that do not follow a normal distribution and the Pearson’s X^2^ test for categorical variables, *p*-values between time points are derived from comparisons between the two ratings using Wilcoxon’s signed rank test for variables not following the normal distribution, and McNemar’s Bowker Test for categorical variables; statistically significant correlations are shown in bold.

## Data Availability

The data presented in this study are available on request from the corresponding author. The data are not publicly available due to privacy restrictions.
